# Long Term Results of Longitudinal Pancreatico-Jejunostomy for Chronic Pancreatic Pain

**DOI:** 10.1155/1996/25261

**Published:** 1996

**Authors:** M. M. Davies, T. O. Oshodi, T. J. Havard, M. H. Lewis

**Affiliations:** Department of Surgery East Glamorgan General Hospital Mid Glamorgan United Kingdom


*HPB Surgery*, 1996, Vol.10, pp.83–86
Reprints available directly from the publisher
Photocopying permitted by license only

© 1996 OPA (Overseas Publishers Association)
Amsterdam B.V. Published in The Netherlands
by Harwood Academic Publishers
Printed in Malaysia

### Long Term Results of Longitudinal Pancreatico-Jejunostomy for Chronic Pancreatic Pain

M. M. DAVIES, T. O. OSHODI, T. J. HAVARD and M. H. LEWIS

Department of Surgery, East Glamorgan General Hospital, Mid Glamorgan

*(Received 1 January1994)*

### INTRODUCTION

Chronic pancreatic pain is frequently severe, recurrent and intractable. It dominates the lives of people that suffer from it. Medical management of this problem primarily involves adequate analgesia (often regular opiates) and treatment of the results of pancreatic dysfunction e.g. steatorrhoea and diabetes mellitus. Advise regarding alcohol consumption is also an important facet of conservative therapy. While this policy may prove successful in a number of patients there is a group who require frequent hospital admission for acute on chronic attacks of pain. When reviewed in the surgical clinic these patients are often seen by the most junior member of the surgical team who advises a reduction in the quantity of both alcohol and analgesia. In reality the patient is usually drinking comparatively little and requires the analgesia for what is intractable pain. Such patients are classified as opiate addicts, "not fit for surgical intervention".

We feel that such patients should be reviewed, not necessarily in a specialist clinic, but by the consultant in charge and preferably when he has an "interest" rather than a "disinterest" in the condition. It is our experience careful selection and treatment of these patients can lead to excellent pain relief, often with the patients returning to work. Although there are a number of treatment options available which include coeliac plexus block, pancreatic resection and drainage procedures, we feel that in those patients with pancreatic duct dilatation a drainage procedure is useful. Frequently, the specific criticism levelled at this procedure is that the pain relief it affords is short lived, it was our impression that this is not necessarily the case.

We therefore present our results of the long term follow up of the eight patients who have undergone a longitudinal pancreatico-jejunostomy for chronic pancreatic pain in a district general hospital.

### PATIENTS AND METHODS

Of a large number of patients with chronic pancreatitis we have selected 8 who have undergone longitudinal pancreatico-jejunostomy for intractable pain over an 8 year period, 1984–1992, in a district general hospital. All patients were operated on by one consultant (MHL). There were 5 males and 3 females with an age range of between 32 and 83 years (mean 59 years).

All patients were assessed initially by interview with the consultant, following this they were investigated with ultrasound, ERCP and CT scan. In each patient a dilated pancreatic duct was identified. At the time of surgery an on table pancreatogram was performed. This is a simple procedure in which the pancreatic duct was cannulated with a 23G Butterfly (Venisystems) needle. Initially, fluid was aspirated from the pancreatic duct and sent for cytology, amylase assay and culture. Approximatley, 10 ml of non-ionic contrast

*Correspondence to*: Mr. M.H. Lewis, MD, FRCS, Consultant General Surgeon, East Glamorgan General Hospital, Mid Glamorgan medium was then injected until there was slight resistance to flow. A plain x-ray film was then taken. This procedure confirmed a dilated pancreatic duct in each of our eight cases before proceding to the pancreatico-jejunostomy. The on-table pancreatogram also allowed visualisation of the morphology of the pancreatic duct. This is important when there are multiple strictures in the duct that will require opening during the operation, prior to the anastomosis.

Following confirmation of the dilated duct with the pancreatogram, the pancreatic duct was opened throughout it's length. A Roux-en-Y loop was then fashioned and anastomosed to the pancreatic duct with multiple interrupted non absorbable stitches. An enteroenterostomy was then fashioned 18in distal to the anastomosis to preserve gastroentestinal continuity. Post-operatively, the patients were routinely admitted to the ITU for observation for a period of 24–48 hours.

A data sheet consisting of several variables was used to obtain information regarding complications as well as pain relief. This information included the subsequent clinic visits following discharge from the wards.

### RESULTS

Longitudinal pancreatico-jejunostomy was performed on eight patients. There was no early post-operative morbidity or mortality in this series. Three of the patients had chronic pancreatitis secondary to gallstones, while in two patients the aetiology was alcohol. One patient had undergone resection for an Apudoma of the head of the pancreas three years prior to the pancreatico-jejunostomy. Two patients had adenocarcinoma of the pancreas but neither of them had been diagnosed pre-operatively.

All patients had immediate relief of their chronic pain, needing opiate analgesia only in the early post-operative period. The pain relief has been continued throughout the period of study and for up to nine years in one patient. Pre-operatively all 8 patients required opiate analgesia and repeated hospital admissions for relief of their pain. In each case the duration of the pain had been prolonged, in one case it had present for 19 years (Range; 4 mths to 19 yrs-Mean 7.5 yrs). Post-operatively, none of the five patients with non-malignant disease require analgesia.

One patient, known to have an Apudoma of the head of the pancreas which had been resected 3 years previously, had a pancreatico-jejunostomy performed for the pain due to pancreatic duct dilatation. Preoperative CT and ultrasound examinations failed to show any evidence of tumor recurrence. Improvement in pain relief post-operatively was immediate and the patient remains pain free five years following surgery.

Of the 5 patients still alive 4 have regained their pre-operative weight, the fifth has increased his weight by 10 kg, an increase of 20% over his pre-operative weight.

One patient continued to have steatorrhoea after the operation, while two patients have developed steatorrhoea between 3 and 4 years post-operatively. All 3 patients symptoms have been successfully controlled with the oral enzyme supplement Creon (Registered Trademark).

One patient, known to be a non-insulin dependent diabetic pre-operatively, remained unchanged. A further patient became diabetic 3.5 years post-surgery. He has been managed with an oral hypoglycaemic agent.

Three patients died during the study period. One died of "natural causes" four months post-operatively, confirmed by post-mortem examination. The other two patients died of metastatic adenocarcinoma of the pancreas 10 months and 18 months post-operatively. These two patients with adenocarcinoma were not diagnosed pre-operatively despite the use of full history and examination, ultrasound, CT and ERCP investigations. At operation one patient was felt to have a suspicious mass in the head of the pancreas and fine needle aspiration biopsy confirmed a carcinoma of the head of the pancreas with a dilated duct. Since the abdomen was open it was decided despite this to continue with the by-pass procedure. This patient was painfree post-operatively but developed further pain just prior to her death presumably due to local invasion. The other patient who died of metastatic adenocarcinoma of the pancreas had a normal fine needle aspiration biopsy of the pancreas at the time of the operation. This patient was pain free postoperatively, she then developed a different type of pain three months after the operation at which time she had evidence of liver metastases on ultrasound scan. This patient died 18 months post-operatively.

### DISCUSSION

The pain and morbidity caused by chronic pancreatitis is severe in most cases, causing many patients to resort to alcohol, opiate drugs and even contemplate suicide[1]. Despite a large number of patients referred, longitudinal pancreatico-jejenostomy was selected for only 8 who were felt to be suitable. All our patients had intractable pain which necessitated regular opiate analgesia. In 2 patients, although alcohol was an aetiological factor, it continued to be taken for pain relief. Unfortunately, most patients get caught up in a viscous circle with increasing demand for opiate analgesia[2] and are therefore felt by surgeons not to warrant their time and effort. They are frequently seen in the follow up clinic by the most junior member of the team and simply advised to stop drinking and booked for further review after the doctor has left the firm.

Although there is some evidence that the pancreatic pain spontaneously burns itself out within five years[3], this is a long time for the patients to have to endure the pain. In our experience most patients were eventually referred for surgical consultation, at which time 4 of our patients had suffered pain for more than 5 years and one for as long as 19 years. In our view this invalidates an expectant or conservative policy.

Before surgical intervention is considered, assessment of the pancreas with the use of a full history and examination, ultrasound, CT scanning and ERCP is important[4] in order to exclude as far as possible any other cause of pancreatic pain such as carcinoma and pseudocyts. In the treatment of chronic pancreatic pain there are a number of options available. Coeliac plexus block is an attractive option as it is a relatively simple procedure. Unfortunately, only about half the patients with pancreatitis gain pain relief and this generally only lasts for a few months[5]. The surgical options available include pancreatic resection or drainage procedures. Longitudinal pancreatico-jejunostomy does not require resection of pancreatic tissue and may be performed by a general surgeon with an interest in the condition.

It is a relatively simple procedure which in our study was not associated with early morbidity or mortality, similar findings have been reported by most authors[1,4,6,7,8]. No specialist equipment is required other than facilities for plain x-ray. As there is no tissue loss there is no sudden loss of endocrine or exocrine function. In the long term, however, there may be loss of function secondary to the underlying pancreatic disease and one of our patients developed non-insulin dependant diabetes mellitus 3 years post-operatively.Drake *et al.*[2] found similar results in their study and he suggest that ductal drainage may slow the onset of diabetes.

For patients with chronic pancreatitis and a dilated pancreatic duct a drainage procedure appears to be an acceptable treatment option. The pain in such patients is thought to be due to raised intraductal pressure[7] and decompression may lead to resolution of symptoms (9). Each of our patients was confirmed to have a dilated pancreatic duct shown by the ultrasound scan, ERCP or CT scan pre-operatively and always a pancreatrogram intra-operatively.

The results of the pancreatico-jejunostomy for relief of chronic pancreatic pan is excellent, both in the short and long term. Of the 5 surviving patients, all have been painfree for a significant period of time, which in one case has lasted for 9 years (Range 16 mths to 9 yrs, Mean 5.5 yrs).

A gradual of loss of exocrine function is indicated by the development of steatorrhoea. This occurred in 3 of our patients but their symptoms were adequately controlled by the use of enzyme supplements (Creon (Duphar)). Despite some loss of pancreatic function, all surviving patients in our study regained and maintained their pre-operative body weight, while in one a gain of 20% was observed. Sato *et al.*[8] had similar findings in their study which found a 5% increase in the weights of 27% of their patients.

In our study, 2 patients were found to have adenocarcinoma of the pancreas which was not diagnosed pre-operatively. Biopsy of the pancreatic tissue at the time of the drainage procedure only detected one of these cases of adenocarcinoma. This highlights the difficulty in excluding coexisting carcinoma in a patient suffering with longstanding pancreatic pain despite extensive investigation. Greenlee *et al.*[10] found three similar patients out of a total of 100 patients. Both of our patients in this situation benefitted from months of pain relief following surgery although their pain did recur and they died of metastatic disease 10 and 18 months post-operatively.

In conclusion, longitudinal pancreaticojejunostomy is a relatively simple procedure associated with minimal morbidity and no mortality with the benefit of longlasting pain relief. It provides an option for treating a disease in which the patient may otherwise be condemed to a life dominated by recurrent episodes of intractable pain leading to addiction to opiate drugs and the risk of suicide.

### REFERENCES

1. Horrow C.E. Cohen J.L, Sutherland D.E.R, Najarian J. S,–Chronic pancreatitis: Long term surgical results of pancreatic duct drainage, pancreatic resection and near total pancreatectomy and islet auto-transplantation (*Surgery* 1984; **96**: 608–14).
2. Drake D.H, Fry W.J. Ductal drainage for chronic pancreatitis (*Surgery* 1989; **105**: 131– 40).
3. Leger L, Lenriot J P, Lemaigre G–Five to twenty year follow up after surgery for chronic pancreatitis in 148 patients. (*Ann.Surg.* 1979; **180**: 185091).
4. Holmberg J T, Isaksson G, Ihse I–Long term results of pancreatico-jejunostomy in chronic pancreatitis. *(Surgery, Gynaecology and Obstetrics* 1985; **160**: 339–46).
5. Leung J W C, Bourne-Wright M, Aveling W, Shorvon P J, Cotton P B–Coeliac block for pain in pancreatic cancer and chronic pancreatitis (*Br J Surg*. 1983; **70**: 730).
6. Morel P, Rohner A –Surgery for chronic pancreatitis (*Surgery* 1987; **101**: 130– 35).
7. Bradley E L–Pancreatic duct pressure in chronic pancreatitis (*AMJ Surg* 1982; **144**: 313–16).
8. Sato T, Miyashita E, Matsuno S, Yamanchi H–The role of surgical treatment of chronic pancreatitis (*Ann Surg* 1986; **203**(3) 266–71).
9. Cooper M J, Williamson R C –Drainage operations in chronic pancreatitis (*Br J Surg* 1984; **71**: 761–66).
10. Greenlee H B, Prinz R A, Aranha G V –Long term results of side to side pancreatico-jejunostomy (World Journal of *Surgery* 1990; **14**: 70–76).

